# Proteomic analysis of PBMCs: characterization of potential HIV-associated proteins

**DOI:** 10.1186/1477-5956-8-12

**Published:** 2010-03-12

**Authors:** Lijun Zhang, Xiaofang Jia, Xiaojun Zhang, Jianjun Sun, Xia Peng, Tangkai Qi, Fang Ma, Lin Yin, Yamin Yao, Chao Qiu, Hongzhou Lu

**Affiliations:** 1 Shanghai Public Health Clinical Center affiliated to Fudan University, Shanghai, 201508, PR China; 2 Neurosurgery, Fuzhou General Hospital, Fuzhou 350025, PR China; 3 Institutes of Biomedical Sciences, Fudan University, Shanghai, 200032, PR China

## Abstract

**Background:**

The human immunodeficiency virus type 1 (HIV-1) pandemic has continued unabated for nearly 30 years. To better understand the influence of virus on host cells, we performed the differential proteome research of peripheral blood mononuclear cells (PBMCs) from HIV-positive patients and healthy controls.

**Results:**

26 protein spots with more than 1.5-fold difference were detected in two dimensional electrophoresis (2DE) gels. 12 unique up-regulated and one down-regulated proteins were identified in HIV-positive patients compared with healthy donors. The mRNA expression of 10 genes was analyzed by real time RT-PCR. It shows that the mRNA expression of talin-1, vinculin and coronin-1C were up-regulated in HIV positive patients and consistent with protein expression. Western blotting analysis confirmed the induction of fragments of vinculin, talin-1 and filamin-A in pooled and most part of individual HIV-positive clinical samples. Bioinformatic analysis showed that a wide host protein network was disrupted in HIV-positive patients.

**Conclusions:**

Together, this work provided useful information to facilitate further investigation of the underlying mechanism of HIV and host cell protein interactions, and discovered novel potential biomarkers such as fragment of vinculin, filamin-A and talin-1 for anti-HIV research.

## Background

The human immunodeficiency virus type 1 (HIV-1) pandemic has continued unabated for nearly 30 years. Globally, there are estimated 33.4 million living individuals in 2008 according to AIDS epidemic update 2009 by the World Health Organization (WHO) http://www.unaids.org/en/KnowledgeCentre/HIVData/EpiUpdate/EpiUpdArchive/2009/default.asp. Current therapies direct against viral proteins. Highly active anti-retroviral therapy has been problematic because of long-term toxicity, inhibitor resistance, and the inability to target persistent reservoirs[1,2]. Thus, there is a need for the comprehensive elucidation of HIV-1-mediated effects on host cellular protein networks and unique protein targets for the design of therapeutic drugs.

Over the past few decades, HIV-mediated effects on host cells have typically involved the study of one gene or one protein at a time, to elucidate their functions in lymphocyte or monocyte biology, signaling pathways or immune system [3-5]. However, the method for one gene or one protein is too slow to elucidate HIV mediated effects on host cells.

The advent of proteomics has expanded the focus on cellular function from examination of gene structure and function to the analysis of their encoded proteins. Analyzing encoded proteins is an important advance because proteins are ultimately responsible for controlling most aspects of cellular function, can be regulated by post-translation modification (PTM), and can not be ascertained from simple analysis of gene transcription or translation. Proteomics has been widely used in the study of pathogenesis, etiology and pathology of infectious disorders such as HIV/HIV-positive, tuberculosis, malaria, measles, and hepatitis as reviewed by LIST EO. *et al*[6] Through proteome research, researchers found some new possible biomarkers for HIV-associated cognitive neurological disorders (HAND) diagnosis, such as Cu/Zn superoxide dismutase (Cu/Zn SOD), new treating targets such as PDI-A3 which were up-regulated after zidovudine (AZT) treatment [7]. Furthermore, great progress was obtained in virus-host cell interaction. For example, HIV replication enhances the production of free fatty acids, low density lipoproteins and many key proteins involved in lipid metabolism[8]. Viral infection down-regulates matrix metalloproteinase 9 (MMP 9) secretion[9]. These observations show the potential power of proteomics for the analysis of cellular proteins involved in diseases.

Peripheral blood mononuclear cells (PBMCs) have been recognized as key players in the innate and adaptive immune responses for their ability to recognize molecular patterns that are typical of microorganisms and by their molecular and functional adaptation to invade pathogens [10,11]. Currently, PBMCs are widely used in research and clinical practice, especially in HIV-positive patients [12]. According to previous reports [13-15] carried in PBMC, human immunodeficiency virus type 1 (HIV-1) infections are associated with functional defects in CD4 T cells, marked by unresponsiveness to T-cell signaling, as well as an increased propensity to mitochondrial membrane potential and apoptosis. Several researches [16-18] have shown that HIV-1 induced a lot of changes in host cellular proteins, including caspase 9 and caspase 3-dependent apoptosis, intracellular transport, oxidative stress, *etc*. However, our knowledge is limited to understand such a complicated virus-host interaction.

In this study, we analyzed HIV-1-mediated effects on host cellular networks through PBMCs proteomics. PBMCs were isolated from EDTA-anticoagulated blood collected from HIV-positive patients or healthy donors by ficoll density centrifugation. Proteins of PBMCs were extracted and separated by two-dimensional gel electrophoresis (2DE). The differentially expressed proteins between HIV-positive and healthy donors were identified by ESI-Ion trap and MALDI-TOF/TOF mass spectrometry. The mRNA expression of 10 genes was analyzed by real time RT-PCR. The expression of talin-1, filamin-A, GNB1 and vinculin was further validated by western blotting analysis. Presented data provided novel insights into HIV and host interaction. Potential new biomarkers were found, including fragment of vinculin for anti-HIV research.

## Methods

### Human subjects

43 HIV-positive patients and 42 healthy donors were enrolled in this study (Table 1), including 9, 22 and 12 from patients and 11, 20 and 11 from healthy donors for two dimensional electrophoresis (2DE), PCR and western blotting (WB), respectively. Patients with HIV infection who did not receive antiviral treatment or immunotherapy in the past 6 months and were followed up at Shanghai Public Health Clinical Center were recruited. All patients met the diagnostic criteria of HIV-positive with CD4 < 350/μL, and negative for hepatitis C virus (HCV) and hepatitis B virus (HBV). Blood samples of healthy donors were the remaining samples after medical tests ordered by the physicians at Shanghai Public Health Clinical Center, and were tested negative for human HIV, HBV and HCV. The study protocol was approved by the local Ethics Committee, and all patients were given a written informed consent.

**Table 1:** Characteristics of patients with HIV and healthy donors

Parameter		Patients			Healthy donors	
	2DE	PCR	WB	2DE	PCR	WB
Number	9	22	12	11	20	11
Age	40.5 ± 10.3	42.1 ± 12.0	44.0 ± 9.4	38.5 ± 11.6	41.3 ± 12.3	41.0 ± 10.1
Female	2	3	4	2	4	4
HIV	Yes			No		
HBV	No			No		
HCV	No			No		
CD4		<350			/	

Selection criteria

1. 18 to 60 years old;

2. Consistent with the diagnostic criteria of HIV infection according to the guidelines of prevention and treatment for HIV-positive in China;

a) The clinical history of HIV-positive;

b) CD4<350/μL;

3. Have not received antiviral treatment or immunotherapy;

4. Negative for other viral infections, including HCV and HBV according to antibody test.

### PBMCs separation and detection

Peripheral whole blood (5 mL) was collected into an EDTA-anticoagulated tube. PBMCs were isolated via density gradient centrifugation using Ficoll-Paque™ Plus (Amersham Pharmacia Biotech) according to the manufacturer's instructions. After ficoll density gradient centrifugation, the upper suspension was removed softly, and the white PBMCS were absorbed and washed twice with PBS by low-speed centrifugations (200 g, 10 min). 90 percent of collected PBMC pellets were frozen at -80°C within 4 h from blood collection for proteomic research. The rest was diluted to 200 μL PBS and used for platelet count through analyzed by Blood Cell Analysis Instrument (Cell-Dyn3200, Abbott, Washington, 98057 USA) using standard clinical detection method which meets US Clinical Laboratory Improvement Amendments of 1988 guidelines for AHA performance standard [19].

### 2DE and image analysis

2DE was performed with the IPGphor system (GE (formerly Amersham Bioscience), USA) and PROTEAN II system (Bio-Rad, Hercules, California), as previously described [20]. Briefly, IPG dry strips (pH 3-10 NL (180 × 30 × 0.5 mm)) was used. Isoelectricfocusing (IEF) was conduced automatically to a total of 52.1 KVh. After equilibration, proteins were separated in 11.5% separation gels with 25 mA/gel constant current. The separation gels were made by us according to the standard protocol described by Gorg et al[21]. Three couples of 2-D gels with protein load of 250 μg were stained by silver nitrate for image analysis. Another couple of 2-D gels with protein load of 1,000 μg were stained with Coomassie brilliant blue (CBB) for protein identification.

For image analysis, Imagemaster 2D software (GE Company, USA) was used according to the manufacturer. Briefly, the individual spot volumes were normalized by dividing their optical density (OD) values by the total OD values of all the spots on the gel to remove the artificial factors. The three parallel gels from HIV-positive or healthy donors were divided into a group respectively, and the matched spots in the three parallel gels were averaged respectively. The threshold defined as the significant change in relative spot volume was at least 1.5-fold comparing the average gels of the HIV-positive with that of healthy donors.

### Trypsin Digestion and Protein identification

The protein spots were manually excised, and in-gel digested as described [20,22]. The digested peptides were analyzed by esquire high capacity ion trap (HCT) mass spectrometer (Bruker, Germany) and MALDI-TOF/TOF (Bruker, Germany). For HCT analysis, the tryptic peptide mixtures were injected onto a C18 μ-precolumn (300 μm id × 5 mm, 5 μm, PepMap™) (LC Packings, Amsterdam, the Netherlands) with a flow rate of 20 μL/min in Ultimate 3000 (Dionex Corporation, USA). After desalted by precolumn, the peptides were eluted to a C-18 reversed-phase nanocolumn (75 μm id × 15 cm length, 3 μm, PepMap™) (LC Packings) using a 3-50% continuous acetonitrile gradient at 300 nL/min. The eluted peptides from the reversed-phase nanocolumn were on line injected to a PicoTip emitter nanospray needle (New Objective, Woburn, MA, USA) for real-time ionization and peptide fragmentation on HCT mass spectrometer.

For MALDI-TOF/TOF analysis, the tryptic peptide mixtures were loaded onto a 384 AnchorChip target (Bruker Daltonik, Bremen, Germany) and analyzed by mass spectrometry according to our published papers [20,22,23].

The MS/MS data was input to MASCOT 2.0 program (MatrixScience, Boston, MA, USA) to search against SwissProt 54.8 identification. Search parameters were set as follows: enzyme, trypsin; allowance for up to one missed cleavage peptide; mass tolerance, 1.2 Da for HCT and 50 ppm for MALDI-TOF/TOF, and MS/MS mass tolerance, 0.6 Da; fixed modification parameter, carbamoylmethylation (C); variable modification parameters, oxidation (at Met); auto hits allowed (only significant hits were report); results format as peptide summary report. Proteins were identified on the basis of peptides whose ions scores exceeded the threshold (p < 0.05), which indicates identification at the 95% confidence level for these matched peptides. For HCT data, proteins identified by more than 4 peptides were accepted and each peptide was manually inspected. Of which, there must be a peptide with four or more continue y-or b-series ions (e.g., y4, y5, y6, y7). For MALDI-TOF/TOF analysis, only proteins with scores over 62 were accepted.

### Data analysis and bioinformatics

The function of the identified proteins was elucidated by SWISS-PROT database http://www.expasy.org and the interaction between the differentially expressed proteins and HIV proteins were checked by HIV interaction database http://www.ncbi.nlm.nih.gov/RefSeq/HIVInteractions. A protein-protein interaction network was drawn by STRING 8.0 http://string.embl.de/ using proteins identified in this work and data from SWISS-PROT function annotation as input. Cytoscape software was used to integrate human proteins with HIV proteins to draw a protein-protein interaction of HIV and host cell proteins.

### RNA extraction and quantitative real-time RT-PCR

Total RNA from PBMCs was extracted using Trizol reagent (Invitrogen Life Technologies) following the manufacturer's instructions and the described previously[5]. Briefly, real-time PCR was performed in the iCycler iQ Multicolor Real-Time PCR Detection System (Bio-Rad Laboratories) using the SYBR Green (ToYoBo). 2 μl cDNA templates was used for each PCR with 0.7 μl 10 nM sense and antisense primers (Table 2) in a total volume of 25 μl. The thermal cycling conditions comprised 3 min at 95°C, followed by 40 cycles at 95°C for 5 s, 60°C for 20 s, and 72°C for 20 s. All of the reactions were performed in duplicate. The threshold cycle of each PCR was converted to a DNA equivalent by reading standard curves generated by amplifying dilutions of a linearized plasmid containing the 188 bp GAPDH cDNA. The relative quantity of the target mRNA was normalized to the level of the internal control GAPDH mRNA level.

**Table 2:** Quantitative analysis results of mRNA expression of 10 differentially expressed proteins in PBMCs from HIV-positive patients and healthy donors.

gene name	strand	primer	Aaymp. Sig.(2-tailed)	Number (HIV/Normal)	Mean peak (HIV/Normal)	Expression of proteins in HIV
KPYM	sense	ctatcctctggaggctgtgc	0.029	10/10	0.6	↑11.9
	antisense	ccagacttggtgaggacgat				
TLN1	sense	tctcccaaaatgccaagaac	0.022	20/22	1.5	↑4.1
	antisense	ctccactagcccttgctgtc				
CAP1	sense	gtgtcaacagccagcagaaa	0.004	10/10	0.8	Only
	antisense	gcggcatcattcatttcttt				
ENOA	sense	gagctccgggacaatgataa	0.631	10/10	1.1	Only
	antisense	tgttccatccatctcgatca				
EHD3	sense	ctaaccctgtgctggagagc	0.009	10/10	0.8	Only
	antisense	gtcagctttgttcagcacca				
COR1C	sense	gcagaagagtggttcgaagg	0.047	20/22	1.4	↑2.0
	antisense	tgatcaggtcgcacttcttg				
ST1A3	sense	catgaaggagaaccccaaaa	0.739	10/10	1.1	↑1.8
	antisense	tgaaggtggtcttccagtcc				
FLNA	sense	aagtgaccgccaataacgac	0.393	10/10	0.8	↑1.7
	antisense	ggcgtcaccctgtgacttat				
VINC	sense	gccaagcagtgcacagataa	0.007	20/22	1.6	↑14.1
	antisense	tctttctaacccagcgcagt				
GNB1	sense	cttgtgatgcttcagccaaa	0.078	20/22	1.4	↓1.5
	antisense	tcagcacgaaggtcaaacag				

Isolated PBMCs from HIV-positive and healthy donors were treated with Trizol regent and RNA was extracted according to the manufacturer's instructions. The primers were obtained through primer 3.0 software analysis. The data were statistic analyzed through Mann-Whitney test.

### Western blotting and densitometry analysis

Fifty micrograms of total protein extracts from pooled and individual samples (12 HIV-positive patients and 8 healthy donors for GNB1, talin-1, vinculin and filamin A) were separated by electrophoresis in SDS-10% polyacrylamide gel and transferred to PVDF membrane (Millipore). After blocking in 10% defatted milk for 2 h, blots were incubated overnight at 4°C with specific primary antibodies (GNB1, 1:2000; talin-1, 1:1000; filamin A, 1:5000, viculin, 1:2000). After three washes with TBS-Tween-20, blots were incubated for 1 h at 20°C with secondary antibody. After further washes, the immune complexes were revealed by enhanced chemiluminescence and detected by X-rays. Each experiment was repeated for three times.

### Statistical Analysis

For all studies, experiments were repeated at least twice. For quantitative data, means and P value were computed. For platelet count and real-time RT-PCR, Mann-Whitney Test was used. For protein spot density in 2DE, a Two-sample t Test software analysis packed in Imagemaster software was used.

## Results

### PBMC analysis

After Ficoll centrifugation, the white blood cell ring fraction (about 2*10^6 ^PBMCs/5 mL whole blood) was collected and used for PBMC quality analysis or proteomic research. To check the contamination of platelets, the blood cell analysis was carried. As shown in Additional file 1, table S1, 25.2 ± 27.8 (average ± std, n = 13) and 24.1 ± 24.4e*10^9^/L (average ± std, n = 11) platelets were detected in the PBMCs from 13 healthy controls and 11 HIV-positive patients respectively. The platelets were reduced for 4 to 12-fold compared with normal reference (100 to 300)*10^9^/L. No statistic difference was found between HIV-positive patients and healthy controls through Mann-Whitney Test statistic analysis (P = 0.34).

### 2-DE and the analysis of gel images

**Figure 1:**
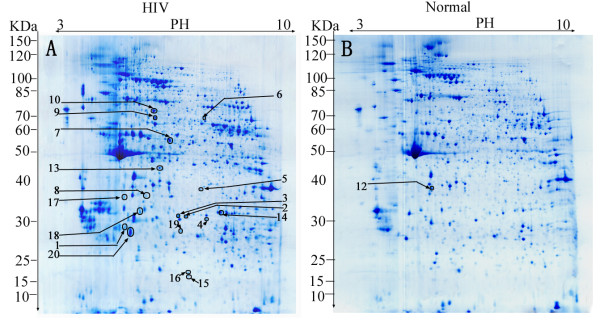
**CBB-stained 2-DE gels of HIV-positive (A) and healthy donors (B)**. Each sample (1000 μg) was subjected to 2DE and CBB staining. Molecular weight of markers is shown on the left. The identified differential proteins (p < 0.05) were marked in gels. Proteins up-regulated in HIV/AIDS were marked in Figure 1A, that down-regulated in Figure 1B.

**Figure 2:**
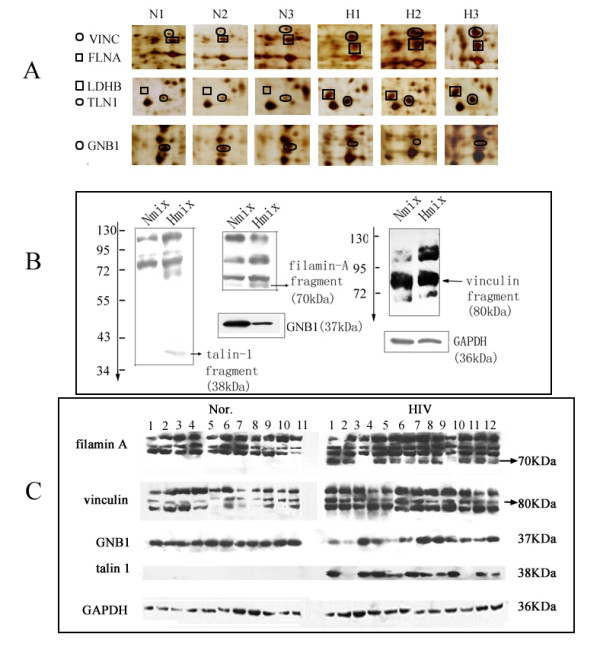
**Western blotting verification of part of the differential proteins**. A. Partially magnified images of five protein spots in 2D gels. B. Western blotting confirmation of talin-1, filamin-A, vinculin and GNB1 in pooled samples. C. Western blotting of GNB1, talin-1, vinculin and filamin-A in 23 individuals (12 from patients and 11 from healthy controls). GAPDH was used as the internal control.

Proteins can be well separated through pH3-10 NL gel stripe in the first dimension and 11.5% separation gel in the second dimension. 1275 ± 242 and 1091 ± 172 protein spots were detected in silver-stained 2DE gels of the HIV-positive and the controls through ImageMaster software analysis and confirmed by manually checking. A typical CBB-stained 2-DE proteome spot pattern of PBMCs from HIV-positive patients and healthy controls was shown in Figure 1. 26 protein spots with 1.5 or more fold difference were detected. The partially magnified images of five protein spots including talin-1(TLN1) (spot 2), filamin-A (FLNA) (spot 9), *etc*. were shown in Figure 2A.

### Protein identification

As shown in Table 3 and Figure 1, 20 differentially expressed spots were successfully identified which corresponding to 13 non-redundant proteins identified by HCT with more than four peptides, four of which were simultaneously identified by MALDI-TOF/TOF (Additional file 2, Table S2). These four proteins identified by both HCT and MALDI-TOF/TOF were talin-1, filamin-A, vinculin and EH domain-containing protein 3 with increased expression in HIV-positive patients compared with healthy controls. According to annotations from UniProt knowledgebase (Swiss-Prot/TrEMBL) and Gene Ontology Database, the identified proteins were involved in various cellular functions including binding (7 proteins), enzyme (4 proteins), signal transduction (2 proteins) and immune response (one protein). In particular, most of the differential proteins (58%) were membrane proteins such as talin-1, filamin-A, *etc*. Furthermore, many different spots were identified to be the products of the same gene such as actin(ACTB), adenylyl cyclase-associated protein 1 (CAP1) and alpha-enolase (ENOA).

**Table 3:** List of the differentially expressed protein spots in 2DE of HIV-positive and healthy donors identified by ESI-MS or both ESI-MS and MALDI-TOF/TOF.

spot^a^	Accession NO.^b^	Protein description	MW^c^	pI	Score^d^	Cov.^e^	Abundance HIV/NOR	Function^f^	Location^g^
1	KPYM_HUMAN	Pyruvate kinase isozymes M1/M2 - Homo sapiens (Human)	58470	7.96	173	15%	↑	enzyme	Cytosol

***2***	***TLN1_HUMAN***	Talin-1 - Homo sapiens (Human)	271766	5.77	44	2%	↑	cell-cell junction	Cell membrane; Cell projection; Cytoplasm; Cytoskeleton; Membrane.

3	LDHB_HUMAN	L-lactate dehydrogenase B chain - Homo sapiens (Human)	36900	5.71	316	30%	↑	enzyme	Cytoplasm.

4, 14, 15	CAP1_HUMAN	Adenylyl cyclase-associated protein 1 - Homo sapiens (Human)	52222	8.27	129	16%	↑	actin binding	Cell membrane; PM

5, 13, 16, 19	ENOA_HUMAN	Alpha-enolase - Homo sapiens (Human)	47481	7.01	217	34%	↑	Enzyme	Cell membrane; Cytoplasm; Membrane; Nucleus.

***6***	***EHD3_HUMAN***	EH domain-containing protein 3 - Homo sapiens (Human)	61971	6.06	285	36%	↑	Binding/transport	Cell membrane; Endosome. Membrane

***7***	***COR1C_HUMAN***	Coronin-1C - Homo sapiens (Human)	53899	6.65	253	18%	↑	Actin-binding/transport	actin cytoskeleton

8	ST1A3_HUMAN	Sulfotransferase 1A3/1A4 - Homo sapiens (Human)	34288	5.68	85	23%	↑	Actin-binding/transport	Cytoplasm

***9***	***FLNA_HUMAN***	Filamin-A - Homo sapiens (Human)	283301	5.7	172	9%	↑	Actin-binding	Cytoplasm; Cytoskeleton; plasma membrane.

***10***	***VINC_HUMAN***	Vinculin - Homo sapiens (Human)	124292	5.5	755	26%	↑	actin binding	Cytoplasm; cytoskeleton; Cell junction; adherens junction; Cell membrane; Peripheral membrane protein;

11	IGKC_HUMAN	Ig kappa chain C region - Homo sapiens (Human)	11773	5.58	101	80%	↑	immuno	extracellular region

12	GNB1_HUMAN	Guanine nucleotide-binding protein G(I)/G(S)/G(T) subunit beta-1 - Homo sapiens (Human)	38151	5.6	69	31%	↓	transducer	

17, 18, 20	ACTB_HUMAN	Actin, cytoplasmic 1	42052	5.29	119	29%	↑	ATP binding	Cytoplasm; cytoskeleton.

^a^Spot no. is the unique number which refers to the labels in Figure 1. Protein spots identified by ESI and MALDI were highlighted by bold and italic.

^b^Accession no. is the MASCOT results of ESI-Ion trap searched from the SWISS-PROT database.

^c ^Molecular weight predicted from database.

^d ^Mascot score was selected identified by HCT.

^e ^Sequence coverage (%) means the number of amino acids spanned by the assigned peptides divided by the sequence length.

^f^Protein function from SWISS-PROT database

^g ^Protein location from SWISS-PROT database

### Protein-protein interaction

**Figure 3:**
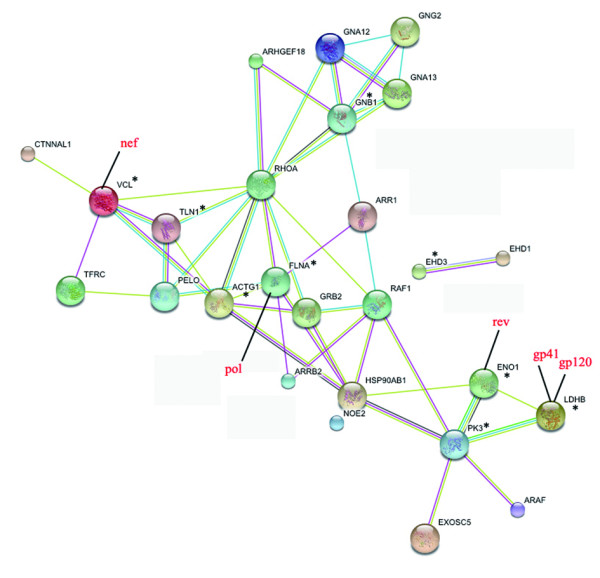
**Protein-protein interactions of identified differential proteins with HIV function proteins**. Proteins labeled with were differentially expressed proteins identified in our work. Proteins labeled in red character were HIV proteins. The rest proteins were host proteins from the database.

As we know, HIV infection is depending on the interaction between HIV proteins and host cell proteins. In this work, bioinformatics analysis was performed to elucidate the network between the identified differential proteins and HIV function proteins. As shown in Figure 3, a wide protein-protein interaction network was affected in HIV/AIDS patients. For example, the host protein interaction net such as VCL - TLN1 - actin, cytoplasmic 2(ACTG1) - FLNA was found to interact with nef and pol (HIV function proteins), and be substantially up-regulated in HIV/AIDS patients. The similar result was observed in ENOA-(L-lactate dehydrogenase B chain) LDHB - pyruvate kinase isozymes M1/M2 (PK3) network which interacting with rev, gp41 and gp120 (HIV function proteins). The related network-protein information is listed in Additional file 3, Table S3.

### Transcriptional profiles of differentially expressed proteins

**Figure 4:**
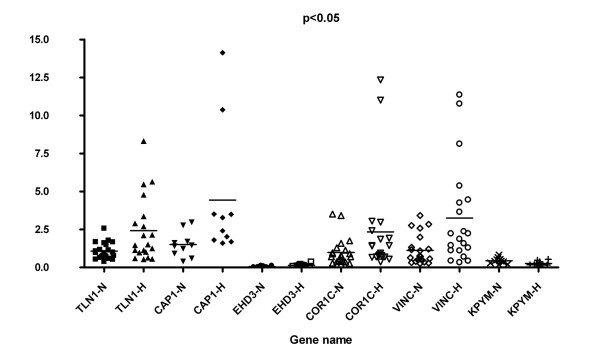
**The mRNA expression of six genes selected from 13 differentially expressed proteins (p < 0.05)**. TLN1-H and TLN1-N represent TLN1 from HIV-positive patients and healthy controls respectively.

In order to make sure whether the protein expression change happens at the transcriptional or translational level, the transcriptional alterations of 10 selected genes in PBMCs from HIV-infected patients (10 or 20) and healthy donors (10 or 22) were measured by quantitative real-time RT-PCR using the mRNA transcript of GAPDH as a control housekeeping gene. As shown in Table 2 and Figure 4, VINC, TLN1, CAP1, pyruvate kinase isozymes M1/M2 (KPYM), EH domain-containing protein 3 (EHD3) and coronin-1C (COR1C) were found to be statistically different between HIV-positive patient and healthy donors. The expression of VINC, TLN1 and COR1C was up-regulated by 1.5, 1.6 and 1.4 fold, respectively, which was consistent with the results from 2DE-MS. In contrast, KPYM has the contrast result with 2DE-MS, down-regulated by 1.7-fold. CAP1 and EHD3 were changed a little with about 0.8 to 1.2- fold differences respectively. FLNA, GNB1, alpha-enolase (ENOA) and sulfotransferase 1A3/1A4 (ST1A3), were found to be no difference between HIV-positive and healthy donors.

### Western blotting (WB)

To further confirm the alterations of protein expression between HIV-positive and healthy donors, four proteins--talin-1, filamin A, vinculin and GNB1 were selected for western blotting analysis through pooled and 23 individual samples including 12 from patients and 11 from healthy controls. For vinculin, a fragment with molecular weight of about 80 KDa was over expressed in pooled and almost all the HIV-positive samples (Figure 2B, 2C), which was consistent with that of 2DE. For talin-1, a fragment with 38 KDa was found in pooled samples from HIV-positive patients; furthermore, this fragment has lower abundance than the other two fragments seen from figure 2B. Through further WB verification (focusing on this fragment) in the 23 individual samples, we found that this fragment could be detected in 10 HIV-positive individuals. However, no positive signal was detected in any healthy controls (figure 2C). For filamin-A, three bands were detected in the samples from healthy donors, while one more band with molecular weight about 70 kDa was found in the pooled and 10 individual samples (Figure 2B, 2C). For GNB1, about 1.8-fold decrease was detected in pooled HIV-1 positive samples. However, GNB1 was found to be down-regulated only in 5 samples of HIV-positive individuals (1, 2, 4, 5 and 11) (Figure 2C). Through statistic analysis, no significant difference (p > 0.05, data not shown) was detected in HIV-positive patients seeing from our study.

## Discussion

The composition of PBMCs may not be constant due to the contamination from red cells and platelets especially platelets. So, it is important to separate PBMCs according to standard protocol. In this work, we separated the PBMCs according to Ficoll-Paque Plus protocol (recommended by Amersham Pharmacia Biotech), and stored them at -80°C within 4 h for proteomic research to decrease artificial difference due to sample treatment and storage. Especially, in order to decrease platelet's contamination, four tips were used, including 1) ficoll density gradient centrifugation; 2) softly removing the upper suspension after gradient centrifugation; 3) only absorbing the white PBMCS fraction; 4) PBS wash for twice using low-speed centrifugations (200 g, 10 min). Due to the above matters, the platelets were not only decreased for 4 to 12 fold, but also no significant difference was detected in two kinds of samples through Mann-Whitey Test statistic analysis of PBMCS from 12 HIV-positive patients and 9 healthy controls. This indicates that the methods reported in this study are reliable and there is no artificial difference during PBMC separation.

For clinical proteomics, it is one of the main considers to decrease the individual variation. In this work, the sample sizes were at least 9. According to the liver proteomic results reported by He Fuchu et al., the individual variation was not significant when the sample size exceeded 7 [20]. Considering the higher individual variation in HIV-infected patients, and referring the HIV-related proteomic research [24-26], the sample size used in work was at least 9. So according to our knowledge, the sample size in this work is basically suitable for proteomic research.

Increasing evidence emphasizes comparative proteomics to screen the differentially expressed proteins associated with HIV/AIDS [6,27]. In our study, we obtained a 2DE profile of the altered protein expression of HIV-positive compared with healthy donors. 13 non-redundant differentially expressed proteins were identified by MS/MS. According to their function annotated in SWISS-PROT http://www.expasy.org, these differentially expressed proteins can be classified into four categories (Table 3).

Seven of the differentially expressed proteins have binding function, which were talin-1, filamin-A, vinculin, coronin-1C, EH domain-containing protein 3, adenylyl cyclase-associated protein 1 and actin. Many studies have shown that the level of talin-1 correlates with HIV infection[28,29]. HIV-1-infected effector T cells binding to primary CD4/CXCR4-targeted T cells results in rapid recruitment to the interface of CD4, CXCR4 and talin [28]. Talin head domain, a fragment of COOH terminus, was reported to be an immunodominant epitope of the antiplatelet antibody response in patients with HIV-1-associated thrombocytopenia[29]. In this study, a new fragment of about 38 kDa was detected. This new fragment might be degraded by HIV [29], or produced during the host cell apoptosis processing for talin involved in cell membrane receptor mediated apoptosis pathway [30] and caspase-mediated cleavage[31]. Vinculin has high affinity to talin-1 [32], and is involved in the attachment of the actin-based microfilaments to the plasma membrane, plays important roles in cell morphology and locomotion [33,34]. Vinculin can be induced to polarizate by HIV-1 Nef [35], were detected to be a caspase-3 target and over-expressed in apoptotic cells [36]. So the fragment detected in this study might be the degradation product of vinculin and can be used as a marker for cell apoptosis in HIV-positive patients. In this work, we also identify another cytoskeleton protein--filamin A, which can interact with pol (a HIV protein), and was reported to be an adaptor protein that links HIV-1 receptors to the actin cytoskeleton remodelling machinery[37]. Filamin A can be cleaved from 280 kDa to 170, 150, and 120 kDa major N-terminal fragments, and 135, 120, and 110 kDa major C-terminal fragments when apoptosis was induced[38]. The 280 kDa representing filamin was decreased in HIV-positive patients [39]. In our research, a fragment with molecular weight of about 70 kDa derived from the COOH terminus of filamin A was increased. So this fragment might be the product of filamin A cleaved by HIV or proteolyzed during apoptosis for filamin are involved in caspase pathway and can be cleaved in HIV-positive patients. These results are consistent with the present knowledge that HIV induces host cell apoptosis through degrading the proteins related to actin cytoskeletal network.

Four of the proteins were enzymes including L-lactate dehydrogenase B chain, pyruvate kinase isozymes M1/M2, adenylyl cyclase-associated protein 1 and sulfotransferase 1A3/1A4. Lactate dehydrogenase B can interact with gp120 and gp41. It was found to be up-regulated by HIV-1 gp120/41 [40]. Pyruvate kinase isozymes M1/M2, a glycolytic enzyme that catalyzes the transfer of a phosphoryl group from phosphoenolpyruvate (PEP) to ADP [41], was found to be down-regulated in HIV-1-infected mouse astrocytes[42] and microglia [16]. However, opposite result was found in PBMCs of HIV-positive patients. This result may be due to 1) the difference between *in vitro *and *in vivo *protein expression, 2) the difference between PBMCs and astrocytes or microglia. Adenylyl cyclase-associated protein 1 is an metabolism enzyme involved in carbohydrate degradation and glycolysis[43]. Sulfotransferase 1A3/1A4 catalyzes the sulfate conjugation of phenolic monoamines, is an important lipid metabolism enzyme[44]. These up-regulated enzymes indicated that a stronger enzyme reaction was induced, and metabolic abnormalities were developed in HIV-infected patients[8,45].

In this work, we also identified three signal transduction proteins. They were GNB1, EH domain-containing protein 3 and coronin-1C. Of which, GNB1 was selected for further verification for it involved as a modulator or transducer in various transmembrane signaling systems[46]. According to the description by Nancy Vazquez [47], the gene expression of GNB1 was found to be up-regulated by 5.0-fold in macrophages infected by HIV-1 compared with uninfected cells. Similarly, Sharon M. Wahl [48] reported that HIV not only induced the up-regulation of GNB1, but the up-regulation of GNB1 could facilitate HIV-1 replication in macrophages. However, we detected this protein was down-regulated in HIV-positive patients analyzed from pooled and part of individual samples. Maybe it is due to the following reasons: 1) Vazquez [47] and Wahl [48] analyzed GNB1 from gene level. However, there are only 50% consistent between mRNA level and protein level. 2) Vazquez [47] and Wahl [48] analyzed GNB1 *in vitro *(HIV-infected macrophages). However, we studied it *in vivo*. Maybe it is due to the difference between *in vitro *and *in vivo*. 3) Vazquez [47] and Wahl [48] reported the up-regulation of GNB1 in macrophages infected by HIV, while we detected the down-regulated in PBMCs including other mononuclear cells as well as macrophages. Further, we only detected the down-regulated expression of GNB1 in part of samples, so there must be some other factors affecting the expression of GNB1. Further researches are needed, such as studying the expression of this protein in different cell groups. We also found one protein-- Ig kappa chain C region related to immune response[49].

According to bio-informatics, these proteins interact with each other as shown in Figure 3. HIV infection affects a wide protein network, some of which were observed before and many were discovered for the first time in this work as discussed above.

It is interesting that most of the identified differential proteins were membrane or membrane-related proteins with binding function although we did not enrich membrane. The up-regulated membrane protein fragment further verified the idea that HIV destructed host cells through degrading the cytokeratin proteins such as talin, filamin A and vinculin[29,50].

## Conclusions

In conclusion, this study adopted a gel-based proteomic approach to probe changed proteins in PBMCs of HIV-positive patients. It is noteworthy that the comparative proteomic approach allowed for the initial identification of 13 altered cellular proteins in HIV-positive patients compared with healthy donors and showed most of the altered cellular proteins are involved in enzyme activation, cell-connection and signaling transduction. A wide protein-protein interaction network was affected in HIV-positive patients. Our study can offer some help in revealing the interactions between HIV and host cell.

## Abbreviations

HIV: human immunodeficiency virus; PBMCs: peripheral blood mononuclear cells; AIDS: acquired immune deficiency syndrome; HAART: high activity antiretroviral therapy; GAPDH: glyceraldehyde-3-phosphate dehydrogenase; HCT: high capacity trap.

## Competing interests

The authors declare that they have no competing interests.

## Authors' contributions

LZ designed the study, participated in statistical analysis of experiment data, interpret the experiment data and draft the manuscript. XJ carried out protein identification. XZ, JS and TQ carried out clinical blood sample collection. XP and CQ carried out PBMCs isolation and 2DE. FM carried out real time RT-PCR and data analysis. LY and YY participated in western blot. HL revised the manuscript critically. All authors read and approved the final manuscript.

## Supplementary Material

Additional file 1 **Table S1 The blood routine analysis results of HIV positive and healthy samples**. No statistical difference of PLA contamination was found between HIV-positive and healthy blood samples Click here for file

Additional file 2 **Table S2 Lists of non-redundant peptides identified in each protein spot**. Lists of non-redundant peptides identified in each protein spot. The first part was identified by ESI-Ion-trap, the second part identified by MALDI-TOF-TOF. Click here for file

Additional file 3 **Table S3 Human proteins and HIV-1 proteins involved in the protein-protein interaction network**. A list of proteins involved in the protein-protein interaction work Click here for file
