# Supplementary material for: Proteomic analysis of PBMCs: characterization of potential HIV-associated proteins
Source: Proteome Sci. 2010 Mar 12;8:12. doi: 10.1186/1477-5956-8-12 (PMC2850332; doi:10.1186/1477-5956-8-12)
Supplement: Additional file 1 — Table S1 The blood routine analysis results of HIV positive and healthy samples. No statistical difference of PLA contamination was found between HIV-positive and healthy blood samples [file 1477-5956-8-12-S1.DOC]

Table S1 The blood routine analyzing PBMCS from 11-HIV positive patients and 13 healthy donors.

|  |  | Cell counting |  |  |  |  |  |  |  |  |  |  |  |  |  |  |
| --- | --- | --- | --- | --- | --- | --- | --- | --- | --- | --- | --- | --- | --- | --- | --- | --- |
| Samples | Name | WBC(10e9/L) | NEU |  | LYM |  | MONO |  | EOS |  | BASO |  | RBC(10e12/L) | HGB | PLT(10e9/L) | MPV() |
| HIV | A734 | 0.943 | 0.019 | 2.06% | 0.474 | 50.2% | 0.411 | 43.6% | 0.002 | 0.242% | 0.037 | 3.87% | 0.005 | 0.00g/L | 96.8 | 6.2 |
| HIV | A735 | 0.367 | 0.016 | 4.45% | 0.328 | 89.3% | 0.019 | 5.04% | 0.00 | 0.00% | 0.004 | 1.19% | 0.002 | 0.00g/L | 35.2 | 5.16 |
| HIV | 142 | 0.29 | 0.003 | 1.12% | 0.274 | 94.4% | 0.012 | 4.09% | 0.001 | 0.372% | 0.00 | 0.00% | 0.00 | 0.00g/L | 2.96 |  |
| HIV | 144 | 0.108 | 0.001 | 0.990% | 0.105 | 97.0% | 0.002 | 1.98% | 0.00 | 0.00% | 0.00 | 0.00% | 0.00 | 0.00g/L | 1.68 |  |
| HIV | A736 | 0.866 | 0.012 | 1.38% | 0.740 | 85.4% | 0.100 | 11.6% | 0.00 | 0.00% | 0.014 | 1.64% | 0.00 | 0.00g/L | 19.3 | 5.79 |
| HIV | N213 | 0.572 | 0.003 | 0.568% | 0.508 | 88.8% | 0.055 | 9.66% | 0.005 | 0.947% | 0.00 | 0.00% | 0.00 | 0.00g/L | 24.2 | 4.91 |
| HIV | A737 | 0.169 | 0.002 | 1.29% | 0.152 | 90.3% | 0.011 | 6.45% | 0.002 | 1.29% | 0.10% | 0.645% | 0.001 | 0.00g/L | 16.5 | 5.86 |
| HIV | N213 | 0.126 | 0.007 | 5.22% | 0.106 | 84.3% | 0.009 | 6.96% | 0.004 | 3.48% | 0.00 | 0.00% | 0.00 | 0.00g/L | 25.0 | 5.04 |
| HIV | 254 | 0.196 | 0.00 | 0.00% | 0.188 | 96.1% | 0.008 | 3.89% | 0.00 | 0.00% | 0.00 | 0.00% | 0.00 | 0.00g/L | 6.20 | 6.49 |
| HIV | HIV003 | 0.529 | 0.001 | 0.205% | 0.518 | 97.9% | 0.010 | 1.85% | 0.00 | 0.00% | 0.00 | 0.00% | 0.00 | 0.00g/L | 2.81 |  |
| HIV | N212 | 0.761 | 0.035 | 4.58% | 0.488 | 64.1% | 0.221 | 29.0% | 0.002 | 0.286% | 0.015 | 2.00% | 0.00 | 0.00g/L | 46.7 | 6.57 |
|  |  | average |  |  |  |  |  |  |  |  |  |  |  |  | 25.2 |  |
|  |  | RSD |  |  |  |  |  |  |  |  |  |  |  |  | 27.8 |  |
| Normal | B124 | 0.724 | 0.004 | 0.579% | 0.639 | 88.3% | 0.080 | 11.0% | 0.00 | 0.00% | 0.001 | 0.145% | 0.00 | 0.00g/L | 12.3 | 7.86 |
| Normal | A121 | 0.499 | 0.007 | 1.47% | 0.435 | 87.2% | 0.051 | 10.3% | 0.00 | 0.00% | 0.005 | 1.05% | 0.00 | 0.00g/L | 9.04 | 6.85 |
| Normal | A139 | 0.159 | 0.002 | 1.31% | 0.138 | 86.9% | 0.018 | 11.1% | 0.00 | 0.00% | 0.001 | 0.654% | 0.00 | 0.00g/L | 0.420 |  |
| Normal | B142 | 0.093 | 0.002 | 2.25% | 0.087 | 93.3% | 0.004 | 4.49% | 0.00 | 0.00% | 0.00 | 0.00% | 0.00 | 0.00g/L | 0.00 |  |
| Normal | B138 | 0.508 | 0.008 | 1.65% | 0.408 | 80.5% | 0.080 | 15.8% | 0.001 | 0.206% | 0.009 | 1.85% | 0.00 | 0.00g/L | 5.890 | 7.25 |
| Normal | B28 | 0.232 | 0.002 | 0.905% | 0.211 | 91.0% | 0.019 | 8.14% | 0.00 | 0.00% | 0.00 | 0.00% | 0.00 | 0.00g/L | 2.40 |  |
| Normal | B21 | 0.557 | 0.015 | 2.63% | 0.455 | 81.8% | 0.081 | 14.5% | 0.002 | 0.376% | 0.004 | 0.752% | 0.001 | 0.00g/L | 10.2 | 8.1 |
| Normal | B41 | 0.528 | 0.008 | 1.58% | 0.405 | 76.6% | 0.099 | 18.8% | 0.002 | 0.396% | 0.014 | 2.57% | 0.00 | 0.00g/L | 12.0 | 5.52 |
| Normal | A136 | 0.827 | 0.014 | 1.65% | 0.727 | 87.9% | 0.078 | 9.40% | 0.00 | 0.00% | 0.008 | 1.02% | 0.001 | 0.00g/L | 5.76 | 7.53 |
| Normal | B131 | 0.437 | 0.001 | 0.240% | 0.379 | 86.6% | 0.055 | 12.5% | 0.00 | 0.00% | 0.003 | 0.719% | 0.00 | 0.00g/L | 3.98 |  |
| Normal | ZLJ | 0.498 | 0.028 | 5.57% | 0.464 | 93.1% | 0.006 | 1.11% | 0.00 | 0.00% | 0.001 | 0.223% | 0.004 | 0.00g/L | 88.3 | 6.3 |
| Normal | CN5 | 1.70 | 0.010 | 0.579% | 1.67 | 98.1% | 0.021 | 1.22% | 0.00 | 0.00% | 0.001 | 0.064% | 0.002 | 0.00g/L | 29.6 | 6.21 |
| Normal | JXF | 0.428 | 0.029 | 6.72% | 0.230 | 53.7% | 0.150 | 35.1% | 0.001 | 0.258% | 0.018 | 4.13% | 0.003 | 0.00g/L | 133 | 7.54 |
|  |  | average |  |  |  |  |  |  |  |  |  |  |  |  | 24.0685 |  |
|  |  | RSD |  |  |  |  |  |  |  |  |  |  |  |  | 24.3964 |  |
|  |  | Mann-Whitney Test (P) |  |  |  |  |  |  |  |  |  |  |  |  | 0.34 |  |
