# Supplementary material for: Proteomic analysis of PBMCs: characterization of potential HIV-associated proteins
Source: Proteome Sci. 2010 Mar 12;8:12. doi: 10.1186/1477-5956-8-12 (PMC2850332; doi:10.1186/1477-5956-8-12)
Supplement: Additional file 2 — Table S2 Lists of non-redundant peptides identified in each protein spot. Lists of non-redundant peptides identified in each protein spot. The first part was identified by ESI-Ion-trap, the second part identified by MALDI-TOF-TOF. [file 1477-5956-8-12-S2.DOC]

Table S2 Lists of non-redundant peptides identified in each protein spot. The first part was identified by ESI-Ion-trap, the second part identified by MALDI-TOF-TOF.

| ***ESI-Ion trap*** | | | | | | | | | | |
| --- | --- | --- | --- | --- | --- | --- | --- | --- | --- | --- |
| Spot 1 | | Pyruvate kinase isozymes M1/M2 | | | | | | | | |
| Observed | | Mr(expt) | Mr(calc) | | Delta | Score | Expect | Rank | Peptide | |
| 599.3200 | | 1196.6254 | 1196.6401 | | -0.0146 | 40 | 0.014 | 1 | R.LDIDSPPITAR.N | |
| 607.2600 | | 1212.5054 | 1212.5696 | | -0.0642 | 45 | 0.0037 | 1 | K.ITLDNAYMEK.C + Oxidation (M) | |
| 680.3500 | | 1358.6854 | 1358.6976 | | -0.0122 | 30 | 0.14 | 1 | R.NTGIICTIGPASR.S | |
| 731.9300 | | 1461.8454 | 1461.8079 | | 0.0376 | 76 | 2.8e-06 | 1 | K.IYVDDGLISLQVK.Q | |
| 818.9000 | | 1635.7854 | 1635.8832 | | -0.0977 | 61 | 8.3e-05 | 1 | K.GVNLPGAAVDLPAVSEK.D | |
| 889.9500 | | 1777.8854 | 1778.8687 | | -0.9832 | 62 | 6.8e-05 | 1 | K.GADFLVTEVENGGSLGSK.K | |
| Spot 2 | | Talin-1 | | | | | | | | |
| 817.4400 | | 1632.8654 | 1632.8392 | | 0.0262 | 26 | 0.24 | 1 | R.VAGSVTELIQAAEAMK.G + Oxidation (M) | |
| 968.4200 | | 1934.8254 | 1934.9109 | | -0.0854 | 25 | 0.27 | 1 | K.EADESLNFEEQILEAAK.S | |
| 770.8000 | | 2309.3782 | 2309.1764 | | 0.2018 | 46 | 0.0016 | 1 | K.VGAIPANALDDGQWSQGLISAAR.M | |
| Spot 3 | | L-lactate dehydrogenase B | | | | | | | | |
| 457.2700 | | 912.5254 | 912.5757 | | -0.0502 | 65 | 4.1e-05 | 1 | K.IVVVTAGVR.Q | |
| 479.3300 | | 956.6454 | 956.6059 | | 0.0396 | 39 | 0.019 | 2 | K.FIIPQIVK.Y | |
| 480.2800 | | 958.5454 | 958.5447 | | 0.0007 | 54 | 0.0006 | 1 | R.GLTSVINQK.L | |
| 386.9500 | | 1157.8282 | 1157.6292 | | 0.1990 | 21 | 0.94 | 1 | K.LKDDEVAQLK.K | |
| 588.7800 | | 1175.5454 | 1175.5822 | | -0.0368 | 79 | 1.9e-06 | 1 | K.SADTLWDIQK.D | |
| 624.8200 | | 1247.6254 | 1247.5928 | | 0.0326 | 56 | 0.00034 | 1 | R.VIGSGCNLDSAR.F | |
| 634.3300 | | 1266.6454 | 1266.6530 | | -0.0075 | 80 | 1.2e-06 | 1 | K.MVVESAYEVIK.L | |
| 815.4500 | | 1628.8854 | 1628.8509 | | 0.0346 | 62 | 7.4e-05 | 1 | K.SLADELALVDVLEDK.L | |
| 655.3600 | | 1963.0582 | 1962.9179 | | 0.1402 | 63 | 0.00014 | 1 | K.GEMMDLQHGSLFLQTPK.I + 2 Oxidation (M) | |
| Spot 4 | | Adenylyl cyclase-associated protein 1 | | | | | | | | |
| 713.9100 | | 1425.8054 | 1425.8079 | | -0.0024 | 33 | 0.037 | 1 | K.LSDLLAPISEQIK.E | |
| 739.7600 | | 1477.5054 | 1477.5966 | | -0.0911 | 22 | 0.47 | 1 | K.EMNDAAMFYTNR.V + Oxidation (M) | |
| 849.9400 | | 1697.8654 | 1697.9028 | | -0.0374 | 71 | 5.6e-06 | 1 | K.AYLSIWTELQAYIK.E | |
| 879.3500 | | 1756.6854 | 1756.8778 | | -0.1923 | 40 | 0.0071 | 1 | R.ALLVTASQCQQPAENK.L | |
| 784.4600 | | 2350.3582 | 2350.2209 | | 0.1373 | 57 | 9.7e-05 | 1 | K.AGAAPYVQAFDSLLAGPVAEYLK.I | |
| Spot 5 | | Alpha-enolase | | | | | | | | |
| 400.6300 | | 799.2454 | 799.3752 | | -0.1298 | 21 | 1.1 | 1 | K.YDLDFK.S | |
| 713.3600 | | 1424.7054 | 1424.7187 | | -0.0133 | 41 | 0.01 | 1 | R.YISPDQLADLYK.S | |
| 508.9100 | | 1523.7082 | 1524.7620 | | -1.0538 | 16 | 2.6 | 1 | K.LAQANGWGVMVSHR.S | |
| 778.8500 | | 1555.6854 | 1555.7705 | | -0.0850 | 90 | 1.2e-07 | 1 | K.VVIGMDVAASEFFR.S + Oxidation (M) | |
| 817.4400 | | 1632.8654 | 1632.8141 | | 0.0513 | 69 | 1.3e-05 | 1 | K.VNQIGSVTESLQACK.L | |
| 647.2700 | | 1938.7882 | 1938.9696 | | -0.1814 | 28 | 0.14 | 1 | K.LAMQEFMILPVGAANFR.E + 2 Oxidation (M) | |
| 678.5800 | | 2032.7182 | 2032.0477 | | 0.6705 | 46 | 0.0017 | 1 | K.FTASAGIQVVGDDLTVTNPK.R | |
| 837.5800 | | 2509.7182 | 2509.1074 | | 0.6108 | 52 | 0.0017 | 1 | K.DYPVVSIEDPFDQDDWGAWQK.F | |
| 1004.6800 | | 3011.0182 | 3010.5625 | | 0.4557 | 19 | 0.4 | 1 | R.HIADLAGNSEVILPVPAFNVINGGSHAGNK.L | |
| Spot 6 | | EH domain-containing protein 3 | | | | | | | | |
| 618.8200 | | 1235.6254 | 1235.6411 | | -0.0156 | 66 | 3.3e-05 | 1 | K.LNAFGNAFLNR.F | |
| 620.7800 | | 1239.5454 | 1239.6322 | | -0.0867 | 29 | 0.16 | 1 | R.VYGALMWSLGK.I + Oxidation (M) | |
| 674.8300 | | 1347.6454 | 1347.6452 | | 0.0002 | 40 | 0.013 | 1 | K.ADQIETQQLMR.V + Oxidation (M) | |
| 692.8300 | | 1383.6454 | 1383.6493 | | -0.0038 | 16 | 3.2 | 5 | R.YLLEQDFPGMR.I + Oxidation (M) | |
| 695.8600 | | 1389.7054 | 1389.7252 | | -0.0197 | 48 | 0.0021 | 1 | K.ELVNNLAEIYGR.I | |
| 697.8500 | | 1393.6854 | 1393.6977 | | -0.0122 | 69 | 1.5e-05 | 1 | K.LDISDEFSEVIK.A | |
| 698.8100 | | 1395.6054 | 1395.6670 | | -0.0616 | 73 | 5.8e-06 | 1 | K.LFEAEEQDLFR.D | |
| 724.8400 | | 1447.6654 | 1447.7195 | | -0.0540 | 33 | 0.059 | 1 | K.DPEVFQTVSEGLK.K | |
| 727.8300 | | 1453.6454 | 1453.6507 | | -0.0053 | 72 | 6.9e-06 | 1 | R.MQDQLQAQDFSK.F + Oxidation (M) | |
| 837.2900 | | 1672.5654 | 1672.7885 | | -0.2231 | 42 | 0.005 | 1 | R.GYDFAAVLEWFAER.V | |
| 671.9600 | | 2012.8582 | 2013.0472 | | -0.1891 | 28 | 0.41 | 1 | R.VYIGSFWSHPLLIPDNR.K | |
| 711.9800 | | 2132.9182 | 2132.9976 | | -0.0794 | 14 | 3 | 2 | R.DKPMYDEIFYTLSPVDGK.I + Oxidation (M) | |
| 809.3600 | | 2425.0582 | 2424.2757 | | 0.7825 | 26 | 0.87 | 1 | K.LLEVVDDMLAHDIAQLMVLVR.Q + 2 Oxidation (M) | |
| 961.7600 | | 2882.2582 | 2882.5099 | | -0.2518 | 51 | 0.00034 | 1 | R.FVCAQLPNPVLESISVIDTPGILSGEK.Q | |
| Spot 7 | | Coronin-1C | | | | | | | | |
| 485.2300 | | 968.4454 | 968.5443 | | -0.0989 | 48 | 0.002 | 1 | R.QLALWNPK.N | |
| 599.7600 | | 1197.5054 | 1197.4720 | | 0.0334 | 34 | 0.051 | 1 | K.NDQCYDDIR.V | |
| 402.8800 | | 1205.6182 | 1205.6669 | | -0.0488 | 58 | 0.00021 | 1 | R.VGIVAWHPTAR.N | |
| 608.2700 | | 1214.5254 | 1214.6151 | | -0.0897 | 54 | 0.00059 | 1 | K.CEPIIMTVPR.K | |
| 672.3700 | | 1342.7254 | 1342.7101 | | 0.0154 | 17 | 2.7 | 1 | R.KCEPIIMTVPR.K | |
| 769.8300 | | 1537.6454 | 1537.6984 | | -0.0529 | 68 | 1.6e-05 | 1 | R.VTWDSSFCAVNPR.F | |
| 858.4000 | | 1714.7854 | 1714.8679 | | -0.0824 | 95 | 3e-08 | 1 | R.AIFLADGNVFTTGFSR.M | |
| 813.9900 | | 2438.9482 | 2439.1270 | | -0.1789 | 57 | 0.00011 | 1 | R.YFEITDESPYVHYLNTFSSK.E | |
| Spot 8 | | Sulfotransferase 1A3/1A4 | | | | | | | | |
| 558.0000 | | 1670.9782 | 1670.8014 | | 0.1767 | 8 | 47 | 2 | R.THPVLYLFYEDMK.E + Oxidation (M) | |
| 596.3900 | | 1786.1482 | 1786.0352 | | 0.1129 | 50 | 0.00072 | 1 | K.SHLPLALLPQTLLDQK.V | |
| 1020.9300 | | 2039.8454 | 2040.0415 | | -0.1961 | 46 | 0.002 | 1 | R.VPFLEVNDPGEPSGLETLK.D | |
| 786.4300 | | 2356.2682 | 2356.1257 | | 0.1425 | 40 | 0.0067 | 1 | K.SGTTWVSQILDMIYQGGDLEK.C + Oxidation (M) | |
| Spot 9 | | Filamin-A | | | | | | | | |
| 483.7600 | | 965.5054 | 965.4454 | | 0.0600 | 30 | 0.14 | 1 | K.AEISFEDR.K | |
| 575.2600 | | 1148.5054 | 1148.6077 | | -0.1023 | 19 | 1.6 | 1 | R.ENGVYLIDVK.F | |
| 631.3600 | | 1260.7054 | 1260.6925 | | 0.0129 | 61 | 9.7e-05 | 1 | R.LTVSSLQESGLK.V | |
| 434.5600 | | 1300.6582 | 1300.6564 | | 0.0017 | 23 | 0.66 | 1 | K.FNGTHIPGSPFK.I | |
| 460.5200 | | 1378.5382 | 1378.6670 | | -0.1288 | 5 | 39 | 4 | K.YGGPYHIGGSPFK.A | |
| 713.8300 | | 1425.6454 | 1425.7463 | | -0.1009 | 81 | 9.6e-07 | 1 | R.EAGAGGLAIAVEGPSK.A | |
| 751.8700 | | 1501.7254 | 1501.7889 | | -0.0634 | 17 | 2.5 | 1 | K.VNQPASFAVSLNGAK.G | |
| 505.9500 | | 1514.8282 | 1514.7518 | | 0.0764 | 14 | 4.6 | 1 | K.FADQHVPGSPFSVK.V | |
| 506.9400 | | 1517.7982 | 1517.7548 | | 0.0433 | 17 | 2.2 | 1 | R.FVPAEMGTHTVSVK.Y + Oxidation (M) | |
| 767.3200 | | 1532.6254 | 1532.7623 | | -0.1369 | 8 | 17 | 1 | R.AEAGVPAEFSIWTR.E | |
| 775.8900 | | 1549.7654 | 1549.7624 | | 0.0031 | 6 | 32 | 8 | R.DAGYGGLSLSIEGPSK.V | |
| 552.2600 | | 1653.7582 | 1653.8145 | | -0.0563 | 36 | 0.025 | 1 | R.APSVANVGSHCDLSLK.I | |
| 667.8000 | | 2000.3782 | 2001.0088 | | -0.6307 | 21 | 0.43 | 1 | K.IPEISIQDMTAQVTSPSGK.T | |
| 734.3300 | | 2199.9682 | 2199.1172 | | 0.8510 | 57 | 0.00072 | 1 | R.LVSNHSLHETSSVFVDSLTK.A | |
| 759.6600 | | 2275.9582 | 2276.0267 | | -0.0685 | 43 | 0.0035 | 1 | K.VHSPSGALEECYVTEIDQDK.Y | |
| 610.7700 | | 2439.0509 | 2439.1819 | | -0.1310 | 16 | 1.5 | 2 | R.VSGQGLHEGHTFEPAEFIIDTR.D | |
| 823.0200 | | 2466.0382 | 2466.1816 | | -0.1434 | 11 | 4.3 | 1 | K.FNEEHIPDSPFVVPVASPSGDAR.R | |
| Spot 10 | | Vinculin | | | | | | | | |
| 469.6600 | | 937.3054 | 937.4505 | | -0.1451 | 37 | 0.027 | 1 | R.DYLIDGSR.G | |
| 501.2500 | | 1000.4854 | 1000.5189 | | -0.0334 | 39 | 0.019 | 1 | K.NQGIEEALK.N | |
| 530.7300 | | 1059.4454 | 1059.4906 | | -0.0452 | 47 | 0.0029 | 1 | K.IAELCDDPK.E | |
| 553.2800 | | 1104.5454 | 1104.6026 | | -0.0572 | 70 | 1.5e-05 | 1 | R.SLGEISALTSK.L | |
| 587.2900 | | 1172.5654 | 1172.6401 | | -0.0746 | 75 | 4.5e-06 | 1 | R.ALASQLQDSLK.D | |
| 592.2700 | | 1182.5254 | 1182.5525 | | -0.0271 | 45 | 0.0039 | 1 | R.LANVMMGPYR.Q + 2 Oxidation (M) | |
| 596.2900 | | 1190.5654 | 1190.5965 | | -0.0310 | 71 | 1e-05 | 1 | K.MSAEINEIIR.V + Oxidation (M) | |
| 615.3200 | | 1228.6254 | 1228.7101 | | -0.0846 | 49 | 0.0019 | 1 | K.ELLPVLISAMK.I + Oxidation (M) | |
| 615.7800 | | 1229.5454 | 1229.5677 | | -0.0222 | 47 | 0.0026 | 1 | R.WIDNPTVDDR.G | |
| 626.3300 | | 1250.6454 | 1250.6363 | | 0.0092 | 66 | 3.1e-05 | 1 | R.VMLVNSMNTVK.E + Oxidation (M) | |
| 657.8500 | | 1313.6854 | 1313.7303 | | -0.0449 | 78 | 2.1e-06 | 1 | K.QVATALQNLQTK.T | |
| 702.8300 | | 1403.6454 | 1403.7144 | | -0.0689 | 79 | 1.4e-06 | 1 | K.ETVQTTEDQILK.R | |
| 729.3600 | | 1456.7054 | 1456.7886 | | -0.0831 | 89 | 1.3e-07 | 1 | K.AQQVSQGLDVLTAK.V | |
| 735.8400 | | 1469.6654 | 1469.6746 | | -0.0092 | 49 | 0.0016 | 1 | R.DPSASPGDAGEQAIR.Q | |
| 739.2800 | | 1476.5454 | 1476.7065 | | -0.1610 | 83 | 5.7e-07 | 1 | K.MLGQMTDQVADLR.A | |
| 742.8700 | | 1483.7254 | 1483.7994 | | -0.0740 | 94 | 5.1e-08 | 1 | R.VDQLTAQLADLAAR.G | |
| 874.9000 | | 1747.7854 | 1747.8417 | | -0.0563 | 109 | 1.2e-09 | 1 | R.VLQLTSWDEDAWASK.D | |
| 664.0300 | | 1989.0682 | 1988.9989 | | 0.0692 | 31 | 0.059 | 1 | K.LVQAAQMLQSDPYSVPAR.D + Oxidation (M) | |
| 1018.4000 | | 2034.7854 | 2035.0473 | | -0.2619 | 8 | 11 | 1 | R.GILSGTSDLLLTFDEAEVR.K | |
| 692.7200 | | 2075.1382 | 2075.1739 | | -0.0357 | 15 | 2.7 | 1 | K.AIPDLTAPVAAVQAAVSNLVR.V | |
| 794.0300 | | 2379.0682 | 2379.1091 | | -0.0409 | 19 | 0.81 | 1 | K.IDAAQNWLADPNGGPEGEEQIR.G | |
| 797.3000 | | 2388.8782 | 2389.0665 | | -0.1884 | 10 | 4.3 | 2 | R.MQEAMTQEVSDVFSDTTTPIK.L + 2 Oxidation (M) | |
| 836.6900 | | 2507.0482 | 2507.2040 | | -0.1559 | 20 | 0.53 | 1 | K.KIDAAQNWLADPNGGPEGEEQIR.G | |
| Spot 11 | | Ig kappa chain C region | | | | | | | | |
| 751.8500 | | 1501.6854 | 1501.7512 | | -0.0657 | 81 | 9.6e-07 | 1 | K.DSTYSLSSTLTLSK.A | |
| 899.8400 | | 1797.6654 | 1796.8880 | | 0.7775 | 28 | 0.13 | 1 | K.SGTASVVCLLNNFYPR.E | |
| 626.0000 | | 1874.9782 | 1874.9197 | | 0.0585 | 52 | 0.00065 | 1 | K.VYACEVTHQGLSSPVTK.S | |
| 649.3300 | | 1944.9682 | 1945.0197 | | -0.0515 | 26 | 0.22 | 1 | -.TVAAPSVFIFPPSDEQLK.S | |
| 712.6500 | | 2134.9282 | 2134.9614 | | -0.0333 | 16 | 2.1 | 1 | K.VDNALQSGNSQESVTEQDSK.D | |
| Spot 12 | | Guanine nucleotide-binding protein G(I)/G(S)/G(T) subunit beta-1 | | | | | | | | |
| 613.3100 | | 1224.6054 | 1224.5809 | | 0.0246 | 34 | 0.051 | 1 | R.LFVSGACDASAK.L | |
| 677.3700 | | 1352.7254 | 1352.6976 | | 0.0278 | 53 | 0.00069 | 1 | K.LIIWDSYTTNK.V | |
| 1008.6500 | | 2015.2854 | 2014.9742 | | 0.3113 | 35 | 0.069 | 1 | K.ACADATLSQITNNIDPVGR.I | |
| 709.6000 | | 2125.7782 | 2126.0143 | | -0.2361 | 20 | 0.63 | 1 | R.LLLAGYDDFNCNVWDALK.A | |
| 811.3400 | | 2430.9982 | 2431.1036 | | -0.1054 | 7 | 12 | 6 | R.VSCLGVTDDGMAVATGSWDSFLK.I + Oxidation (M) | |
| 906.4000 | | 2716.1782 | 2715.2520 | | 0.9262 | 6 | 14 | 6 | R.ADQELMTYSHDNIICGITSVSFSK.S | |
| Spot 13 | | Alpha-enolase | | | | | | | | |
| 400.7100 | | 799.4054 | 799.3752 | | 0.0302 | 21 | 1.3 | 1 | K.YDLDFK.S | |
| 450.2800 | | 898.5454 | 898.5488 | | -0.0033 | 11 | 12 | 7 | K.TIAPALVSK.K | |
| 504.2300 | | 1006.4454 | 1006.4940 | | -0.0485 | 31 | 0.11 | 1 | K.SCNCLLLK.V | |
| 640.8000 | | 1279.5854 | 1279.5788 | | 0.0067 | 16 | 2.8 | 1 | K.LMIEMDGTENK.S | |
| 703.8300 | | 1405.6454 | 1405.7089 | | -0.0635 | 56 | 0.00029 | 1 | R.GNPTVEVDLFTSK.G | |
| 713.3600 | | 1424.7054 | 1424.7187 | | -0.0133 | 51 | 0.001 | 1 | R.YISPDQLADLYK.S | |
| 778.8700 | | 1555.7254 | 1555.7705 | | -0.0450 | 77 | 2.4e-06 | 1 | K.VVIGMDVAASEFFR.S + Oxidation (M) | |
| 902.9600 | | 1803.9054 | 1803.9366 | | -0.0312 | 28 | 0.15 | 1 | R.AAVPSGASTGIYEALELR.D | |
| 647.3500 | | 1939.0282 | 1938.9696 | | 0.0586 | 41 | 0.0067 | 1 | K.LAMQEFMILPVGAANFR.E + 2 Oxidation (M) | |
| 1004.7600 | | 3011.2582 | 3010.5625 | | 0.6957 | 18 | 0.67 | 1 | R.HIADLAGNSEVILPVPAFNVINGGSHAGNK.L | |
| Spot 14 | | Adenylyl cyclase-associated protein 1 | | | | | | | | |
| 713.8900 | | 1425.7654 | | 1425.8079 | -0.0424 | 34 | 0.034 | 1 | K.LSDLLAPISEQIK.E | |
| 731.7200 | | 1461.4254 | | 1461.6017 | -0.1762 | 27 | 0.16 | 1 | K.EMNDAAMFYTNR.V | |
| 879.4500 | | 1756.8854 | | 1756.8778 | 0.0077 | 22 | 0.38 | 2 | R.ALLVTASQCQQPAENK.L | |
| 784.1700 | | 2349.4882 | | 2350.2209 | -0.7327 | 16 | 1.1 | 1 | K.AGAAPYVQAFDSLLAGPVAEYLK.I | |
| Spot 15 | | Adenylyl cyclase-associated protein 1 | | | | | | | | |
| 435.7700 | | 869.5254 | | 870.5175 | -0.9920 | 8 | 21 | 1 | K.VPTISINK.T | |
| 542.7900 | | 1083.5654 | | 1083.5812 | -0.0157 | 26 | 0.37 | 1 | K.EPAVLELEGK.K | |
| 691.7000 | | 2072.0782 | | 2072.0273 | 0.0509 | 55 | 0.00022 | 1 | R.VENQENVSNLVIEDTELK.Q | |
| 938.0300 | | 2811.0682 | | 2810.3109 | 0.7573 | 25 | 0.67 | 1 | K.SSEMNVLIPTEGGDFNEFPVPEQFK.T | |
| Spot 16 | | Alpha-enolase | | | | | | | | |
| 713.3600 | | 1424.7054 | | 1424.7187 | -0.0133 | 28 | 0.19 | 1 | R.YISPDQLADLYK.S | |
| 817.4200 | | 1632.8254 | | 1632.8141 | 0.0113 | 19 | 1.4 | 1 | K.VNQIGSVTESLQACK.L | |
| 1016.8700 | | 2031.7254 | | 2032.0477 | -0.3223 | 9 | 7.7 | 7 | K.FTASAGIQVVGDDLTVTNPK.R | |
| 785.1700 | | 2352.4882 | | 2352.1519 | 0.3363 | 40 | 0.019 | 1 | R.SGETEDTFIADLVVGLCTGQIK.T | |
| 837.4200 | | 2509.2382 | | 2509.1074 | 0.1308 | 74 | 2.6e-06 | 1 | K.DYPVVSIEDPFDQDDWGAWQK.F | |
| Spot 17 | | Actin, cytoplasmic 1 | | | | | | | | |
| 507.7100 | | 1013.4054 | | 1013.4739 | -0.0685 | 22 | 0.91 | 1 | R.DLTDYLMK.I + Oxidation (M) | |
| 566.7500 | | 1131.4854 | | 1131.5197 | -0.0342 | 43 | 0.0082 | 1 | R.GYSFTTTAER.E | |
| 589.3300 | | 1176.6454 | | 1176.6060 | 0.0394 | 40 | 0.016 | 1 | K.EITALAPSTMK.I + Oxidation (M) | |
| 505.9300 | | 1514.7682 | | 1514.7419 | 0.0263 | 21 | 0.88 | 1 | K.IWHHTFYNELR.V | |
| 506.2400 | | 1515.6982 | | 1515.6954 | 0.0028 | 19 | 1.4 | 1 | K.QEYDESGPSIVHR.K | |
| 895.9100 | | 1789.8054 | | 1789.8846 | -0.0792 | 57 | 0.00018 | 1 | K.SYELPDGQVITIGNER.F | |
| 652.0200 | | 1953.0382 | | 1953.0571 | -0.0189 | 60 | 9e-05 | 1 | R.VAPEEHPVLLTEAPLNPK.A | |
| 856.0800 | | 2565.2182 | | 2565.1614 | 0.0567 | 31 | 0.053 | 1 | K.LCYVALDFEQEMATAASSSSLEK.S + Oxidation (M) | |
| Spot 18 | | Actin, cytoplasmic 1 | | | | | | | | |
| 566.7200 | | 1131.4254 | | 1131.5197 | -0.0942 | 34 | 0.054 | 1 | R.GYSFTTTAER.E | |
| 581.3000 | | 1160.5854 | | 1160.6111 | -0.0256 | 41 | 0.012 | 1 | K.EITALAPSTMK.I | |
| 506.2200 | | 1515.6382 | | 1515.6954 | -0.0572 | 33 | 0.061 | 1 | K.QEYDESGPSIVHR.K | |
| 652.0400 | | 1953.0982 | | 1953.0571 | 0.0411 | 40 | 0.0077 | 1 | R.VAPEEHPVLLTEAPLNPK.A | |
| 738.9300 | | 2213.7682 | | 2214.0627 | -0.2945 | 18 | 0.86 | 2 | K.DLYANTVLSGGTTMYPGIADR.M | |
| 855.9800 | | 2564.9182 | | 2565.1614 | -0.2433 | 10 | 3.8 | 2 | K.LCYVALDFEQEMATAASSSSLEK.S + Oxidation (M) | |
| Spot 19 | | Alpha-enolase | | | | | | | | |
| 713.3200 | | 1424.6254 | | 1424.7187 | -0.0933 | 41 | 0.0089 | 1 | R.YISPDQLADLYK.S | |
| 514.5700 | | 1540.6882 | | 1540.7569 | -0.0687 | 3 | 54 | 6 | K.LAQANGWGVMVSHR.S + Oxidation (M) | |
| 778.8500 | | 1555.6854 | | 1555.7705 | -0.0850 | 104 | 4.8e-09 | 1 | K.VVIGMDVAASEFFR.S + Oxidation (M) | |
| 817.3000 | | 1632.5854 | | 1632.8141 | -0.2287 | 61 | 6.4e-05 | 1 | K.VNQIGSVTESLQACK.L | |
| 678.3000 | | 2031.8782 | | 2032.0477 | -0.1695 | 18 | 1.3 | 1 | K.FTASAGIQVVGDDLTVTNPK.R | |
| Spot 20 | | Actin, cytoplasmic 1 | | | | | | | | |
| 507.6700 | | 1013.3254 | | 1013.4739 | -0.1485 | 51 | 0.00088 | 1 | R.DLTDYLMK.I + Oxidation (M) | |
| 566.7300 | | 1131.4454 | | 1131.5197 | -0.0742 | 50 | 0.0014 | 1 | R.GYSFTTTAER.E | |
| 581.2900 | | 1160.5654 | | 1160.6111 | -0.0456 | 63 | 7.3e-05 | 1 | K.EITALAPSTMK.I | |
| 506.2000 | | 1515.5782 | | 1515.6954 | -0.1172 | 49 | 0.0012 | 1 | K.QEYDESGPSIVHR.K | |
| 597.6000 | | 1789.7782 | | 1789.8846 | -0.1065 | 43 | 0.005 | 1 | K.SYELPDGQVITIGNER.F | |
| 744.3200 | | 2229.9382 | | 2230.0576 | -0.1194 | 44 | 0.0032 | 1 | K.DLYANTVLSGGTTMYPGIADR.M + Oxidation (M) | |
| **MALDI-TOF** | | | | | | | | | | |
| spot | accession | | | protein name | | MW | score | matched | | unmatched |
| Spot 6 | IPI00749074 | | | EH domain-containing protein 3 | | 61858 | 73 | 8 | | 11 |
| Spot 7 | IPI00010133 | | | Coronin-1C | | 51678 | 63 | 6 | | 11 |
| Spot 9 | IPI00657767 | | | Filamin-A | | 33474 | 62 | 5 | | 11 |
| Spot 10 | IPI00291175 | | | Vinculin | | 117220 | 129 | 12 | | 10 |
