# Supplementary material for: Proteomic analysis of PBMCs: characterization of potential HIV-associated proteins
Source: Proteome Sci. 2010 Mar 12;8:12. doi: 10.1186/1477-5956-8-12 (PMC2850332; doi:10.1186/1477-5956-8-12)
Supplement: Additional file 3 — Table S3 Human proteins and HIV-1 proteins involved in the protein-protein interaction network. A list of proteins involved in the protein-protein interaction work [file 1477-5956-8-12-S3.DOC]

Table S3 Human proteins and HIV-1 proteins involved in the protein-protein interaction network.

| **Abbreviations** | **Protein name** | **Annotation** |
| --- | --- | --- |
| **Human proteins** | | |
| ACTG1 | Actin, cytoplasmic 2 (Gammaactin) | Detected |
| ARAF | ARaf protooncogene serine/threonineprotein kinase (EC 2.7.11.1) (A raf1) (Protooncogene Pks) | Database |
| ARHGEF18 | Rhospecific guanine nucleotide exchange factor p114 | database |
| ARR1 | Betaarrestin1 (Arrestin beta 1) | database |
| ARRB2 | Betaarrestin2 (Arrestin beta 2) | database |
| EHD1 | EH domaincontaining protein 1 (Testilin) (hPAST1) | detected |
| EHD3 | EH domaincontaining protein 3 | database |
| ENO1 | Alphaenolase (EC 4.2.1.11) (2phosphoDglycerate hydrolyase) (Non neural enolase) (NNE) (Enolase 1) (Phosphopyruvate hydratase) (Cmyc promoterbinding protein) (MBP1) (MPB1) (Plasminogenbinding protein) | detected |
| EXOSC5 | Exosome complex exonuclease RRP46 (EC 3.1.13.) (Ribosomal RNA processing protein 46) (Exosome component 5) (p12B) (Chronic myelogenous leukemia tumor antigen 28) | database |
| FLNA | FilaminA (Alphafilamin) (Filamin1) (Endothelial actinbinding protein) (Actinbinding protein 280) (ABP280) (Nonmuscle filamin) | detected |
| GNA12 | Guanine nucleotidebinding protein alpha12 subunit (G alpha12) | database |
| GNA13 | Guanine nucleotidebinding protein alpha13 subunit (G alpha13) | Database |
| GNB1 | Guanine nucleotidebinding protein G(I)/G(S)/G(T) subunit beta 1 (Transducin beta chain 1) | Detected |
| GNGT1 | Guanine nucleotidebinding protein G(T) gammaT1 subunit precursor (Transducin gamma chain) | Database |
| GRB2 | Growth factor receptorbound protein 2 (Adapter protein GRB2) (SH2/SH3 adapter GRB2) (Protein Ash) | Database |
| HSP90AB1 | Heat shock protein HSP 90beta (HSP 84) (HSP 90) | Database |
| LDHB | Llactate dehydrogenase B chain (EC 1.1.1.27) (LDHB) (LDH heart subunit) (LDHH) (Renal carcinoma antigen NYREN46) | Detected |
| NOE2 | Noelin2 precursor (Olfactomedin2) | Database |
| PELO | Integrin alpha1 precursor (Laminin and collagen receptor) (VLA1) (CD49a antigen) | Database |
| PK3 | Pyruvate kinase isozymes M1/M2 (EC 2.7.1.40) (Pyruvate kinase muscle isozyme) (Pyruvate kinase 2/3) (Cytosolic thyroid hormonebinding protein) (CTHBP) (THBP1) | Detected |
| RAF1 | RAF protooncogene serine/threonineprotein kinase (EC 2.7.11.1) (Raf 1) (CRAF) (cRaf) | Database |
| RHOA | Transforming protein RhoA precursor (H12) | Database |
| TFRC | Transferrin receptor protein 1 (TfR1) (TR) (TfR) (Trfr) (CD71 antigen) (T9) (p90) |  |
| TLN1 | Talin1 |  |
| VCL | Vinculin (Metavinculin) |  |
| **HIV-1 proteins** | | |
| gp120 | Envelope surface glycoprotein gp120 |  |
| gp41 | gp41, Envelope Transmembrane Protein |  |
| nef | Nef |  |
| pol | retropepsin |  |
| ref | Tat |  |
